# Influence of PRRSV-1 vaccination and infection on mononuclear immune cells at the maternal-fetal interface

**DOI:** 10.3389/fimmu.2022.1055048

**Published:** 2022-11-08

**Authors:** Melissa R. Stas, Heinrich Kreutzmann, Julia Stadler, Elena L. Sassu, Kerstin H. Mair, Michaela Koch, Christian Knecht, Maria Stadler, Marlies Dolezal, Gyula Balka, Marianne Zaruba, Marlene Mötz, Armin Saalmüller, Till Rümenapf, Wilhelm Gerner, Andrea Ladinig

**Affiliations:** ^1^ University Clinic for Swine, Department for Farm Animals and Veterinary Public Health, University of Veterinary Medicine Vienna, Vienna, Austria; ^2^ Clinic for Swine, Centre for Clinical Veterinary Medicine, Ludwig-Maximilians-University Munich, Oberschleissheim, Germany; ^3^ Department of Pathobiology, Institute of Immunology, University of Veterinary Medicine Vienna, Vienna, Austria; ^4^ Department of Pathobiology, Christian Doppler Laboratory for Optimized Prediction of Vaccination Success in Pigs, Institute of Immunology, University of Veterinary Medicine Vienna, Vienna, Austria; ^5^ Platform for Bioinformatics and Biostatistics, Department of Biomedical Sciences, University of Veterinary Medicine, Vienna, Austria; ^6^ Department of Pathology, University of Veterinary Medicine Budapest, Budapest, Hungary; ^7^ Department of Pathobiology, Institute of Virology, University of Veterinary Medicine Vienna, Vienna, Austria

**Keywords:** PRRSV, porcine maternal-fetal interface, NK cells, γδ T cells, B cells, CD4 T cells, CD8 T cells

## Abstract

Porcine reproductive and respiratory syndrome virus (PRRSV) is one of the most devastating viruses for the global swine industry. Infection during late gestation causes reproductive failure but the local immune response *in utero* remains poorly understood. In this study, an experimental PRRSV-infection model with two different PRRSV-1 field isolates was used to investigate the immune cell phenotypes at the maternal-fetal interface during late gestation. In addition, phenotypic changes induced by a modified live virus (MLV, ReproCyc^®^ PRRS EU) vaccine were studied. Vaccinated (n = 12) and non-vaccinated pregnant gilts (n = 12) were challenged with either one of the PRRSV-1 field isolates (low vs. high virulent, LV or HV) or sham-inoculated at day 84 of gestation. Twenty-one days post infection all gilts were euthanized and the fetal preservation status for all fetuses per litter was assessed. Leukocytes from the maternal-fetal interface were isolated and PRRSV-induced changes were investigated using *ex vivo* phenotyping by flow cytometry. PRRSV load in tissue from the maternal endometrium (ME) and fetal placenta (FP) was determined by RT-qPCR. In the ME, a vast increase in CD8β T cells with CD8α^pos^CD27^dim^ early effector phenotype was found for fetuses from the non-vaccinated LV and HV-challenged gilts, compared to non-treated and vaccinated-only controls. HV-challenged fetuses also showed significant increases of lymphocytes with effector phenotypes in the FP, including NKp46^pos^ NK cells, CD8α^high^ γδ T cells, as well as CD8α^pos^CD27^pos/dim^ CD4 and CD8 T cells. In vaccinated animals, this common activation of effector phenotypes was more confined and the fetal preservation status significantly improved. Furthermore, a negative correlation between the viral load and CD163^high^CD169^pos^ mononuclear phagocytic cells was observed in the FP of HV-infected animals. These results suggest that the strong expansion of effector lymphocytes in gilts that were only infected causes immune-pathogenesis rather than protection. In contrast, the attenuated MLV seems to dampen this effect, yet presumably induces memory cells that limit reproductive failure. This work provides valuable insights into changes of local immune cell phenotypes following PRRSV vaccination and infection.

## 1 Introduction

Porcine reproductive and respiratory syndrome virus (PRRSV), belonging to the *Arteriviridae* family (1), is the cause of PRRS which has a massive negative economic impact on global swine industry (2–4). This enveloped, positive-stranded RNA virus preferentially infects cells of the monocytic lineage (1, 5); however, some dendritic cell populations have also been shown to be permissive for viral replication *in vitro* (6). PRRSV exists in two genetically distinct species, *Betaarterivirus suid 1* (PRRSV-1) and *Betaarterivirus suid 2* (PRRSV-2) (7–9). Between and within species, a high degree of genetic diversity has been described (10, 11), which might explain observed differences in virulence and severity of clinical outcome (12, 13). A high mutation rate and genetic recombination events contribute to PRRSV heterogeneity (11) and inevitably have repercussions on vaccine efficacy and design.

Modified live virus (MLV) vaccines are widely used as a preventive or therapeutic measure to mitigate clinical signs, financial losses and transmission of the virus. These vaccines are considered efficacious, especially when compared to killed vaccines (14), but no clear correlates of protection have been identified so far (5, 15). The PRRSV-specific antibody responses that occur early after infection are non-neutralizing and do not correlate with clinical protection (5, 15). Neutralizing antibodies (NAbs) occur late (about four weeks post infection) and can confer protection (5, 15). NAbs are mostly strain specific, although heterologous NAbs have been identified (16, 17). Interferon-γ (IFN-γ) producing T cells and NK cells are considered to be involved in protection (5, 15, 18–21). Furthermore, a recent study showed that local T cell responses in the lung are already induced ten days post infection (dpi) and seem to be linked to viral clearance (20).

As to date, several molecules have been implicated as potential receptors for PRRSV including: CD163, CD169 (also known as sialoadhesin or siglec-1), non-muscle myosin heavy chain 9, heparin sulfate, CD151, vimentin, and DC-SING (CD209) (22). The cysteine-rich scavenger receptor CD163 is considered as the main receptor for PRRSV internalization and disassembly (5, 22) as pigs with a complete CD163 knock-out are resistant to PRRSV infection (23). CD169 is considered as a co-receptor which may assist in viral attachment/internalization but is not a requirement to establish a PRRSV infection (5, 22). Momentarily, CD163 and CD169 are the most extensively studied. The potential role of the other mentioned co-receptors in context of PRRSV is reviewed here (22).

The reproductive form of PRRS is associated with transplacental infection of the fetuses and primarily occurs during late gestation (24–26). This might be related to the frequency of CD169^pos^ cells located at the maternal-fetal interface (27). An epithelial bilayer sequesters the porcine maternal-fetal interface and is considered as a tight, impermeable barrier (28). The mechanisms responsible for reproductive failure remain elusive, although several hypotheses exist (26, 29–32). Currently, it is thought that post-infection events at the maternal-fetal interface are the cause for fetal deterioration and demise (33–36).

We recently described lymphocyte phenotypes that reside at the maternal-fetal interface in healthy sows during late gestation (37). More NKp46^pos^ and NKP46^neg^ NK cells were identified in the maternal endometrium (ME) and fetal placenta (FP), compared to fetal spleens. In the FP, however, also NKp46^high^ NK cells were found. CD4, CD8, and γδ T cells in the ME predominantly exhibited differentiated effector phenotypes whereas in the FP naive phenotypes prevailed. Investigations concerning the PRRSV-mediated immune response at the maternal-fetal interface are limited. Following infection, PRRSV-infected monocytes reach the endometrium *via* the endometrial vessels (5, 26). Hereafter, the virus replicates in CD163^pos^CD169^pos^ macrophages and causes apoptosis of infected cells and bystander cells (5, 26, 33). Ten dpi a higher number of these virus susceptible cells are found in the ME and FP of PRRSV-infected sows (33). Furthermore, an increase in CD3^neg^CD8α^pos^ cells was also found in the ME of PRRSV-infected animals through immunofluorescence staining (33).

Due to these limited findings, we investigated local changes in immune cell phenotypes at the maternal-fetal interface in response to two PRRSV-1 field isolates, using *ex vivo* phenotyping by flow cytometry. With the same methodology, we also investigated the influence of a PRRSV-1 MLV (ReproCyc^®^ PRRS EU) immunization prior to challenge infection, which was previously shown to partially prevent vertical transmission following heterologous PRRSV-1 AUT15-33 infection (38).

## 2 Material and methods

### 2.1 Animals and experimental design

Twenty-four healthy crossbred (Landrace × Large White) gilts were purchased from a specialized producer (PIC Deutschland GmbH) and housed in a commercial Austrian piglet-producing farm free of PRRSV, as confirmed by regular serological monitoring. All gilts were vaccinated against porcine parvovirus 1 in combination with *Erysipelothrix rhusiopathiae*, swine influenza A virus, and porcine circovirus type 2, as previously described (38). Prior to insemination (142 and 114 days prior to infection) and during mid-gestation (31 days prior to infection), twelve randomly selected gilts were vaccinated with a PRRSV MLV vaccine (ReproCyc^®^ PRRS EU, Boehringer Ingelheim Vetmedica GmbH, Ingelheim am Rhein, Germany) according to the instructions of the manufacturer. Vaccinated and non-vaccinated gilts were housed separately but under identical housing conditions. At day 77/78 of gestation, vaccinated and non-vaccinated gilts were relocated to a biosafety level 2 unit of the University of Veterinary Medicine Vienna on two consecutive days. All gilts were randomly allocated into six groups: 1. non-vaccinated and non-infected, No.Vac_No.Chall; 2. vaccinated and non-infected, Vac_No.Chall; 3. non-vaccinated and infected with low virulent (LV) strain, No.Vac_Chall_LV; 4. vaccinated and infected with low virulent (LV) strain, Vac_Chall_LV; 5. non-vaccinated and infected high virulent (HV) strain, No.Vac_Chall_HV; 6. vaccinated and infected with high virulent (HV) strain, Vac_Chall_HV (n = 4/group). Each group was housed in individual rooms with isolated airspaces. After one-week of acclimation, experimental infection was performed as described previously (39). Eight gilts (4 vaccinated and 4 non-vaccinated) were inoculated intranasally and intramuscularly (50% IN, 50% IM), with an infectious dose of 3 × 10^5^ TCID_50_, with either one of two different PRRSV-1 field isolates (LV or HV) or sham-inoculated with cell culture medium (DMEM, Thermo Fischer Scientific, Carlsbad, CA, United States) at day 84 of gestation. An overview of the six groups is given in **Table 1**. All experiments were approved by institutional ethics and animal welfare committee (Vetmeduni Vienna) and the national authority according to §§26ff. of Animal Experiments Act, Tierversuchsgesetz 2012 – TVG 2012 (GZ 68.205/0142-WF/V/3b/2016).

**Table 1:** Overview six treatment groups.

Groups	n	Vaccination PRRSV^*^	Infection PRRSV^**^
No.Vac_No.Chall	4	–	–
Vac_No.Chall	4	3 doses Reprocyc^®^ PRRS EU	–
No.Vac_Chall_LV	4	–	LV dog 84, 50% IN + 50% IM
Vac_Chall_LV	4	3 doses Reprocyc^®^ PRRS EU	LV dog 84, 50% IN + 50% IM
No.Vac_Chall_HV	4	–	HV dog 84, 50% IN + 50% IM
Vac_Chall_HV	4	3 doses Reprocyc^®^ PRRS EU	HV dog 84, 50% IN + 50% IM

No.Vac_No.Chall, non-vaccinated and non-infected; Vac_No.Chall, vaccinated and non-infected; No.Vac_Chall_LV, non-vaccinated and infected with low virulent (LV) strain; Vac_Chall_LV, vaccinated and infected with low virulent (LV) strain; No.Vac_Chall_HV; non-vaccinated and infected high virulent (HV) strain; Vac_Chall_HV, vaccinated and infected with high virulent (HV) strain.

LV, low virulent; HV, high virulent; dog, day of gestation; IN, intranasal; IM, intramuscular.

^*^2 Reprocyc^®^ PRRS EU doses prior to insemination and 1 dose mid-gestation.

**PRRSV infection dose 3 × 10^5^ TCID_50_.

### 2.2 Virus isolates for challenge

Two European PRRSV-1 field isolates with a documented history of reproductive pathogenesis, as communicated by veterinarians in the field, were used. The PRRSV-1 field isolate 720789 (Genbank Accession number OP529852, kindly provided by Christoph Keller, Boehringer Ingelheim Vetmedica GmbH), further referred to as the ‘low virulent strain (LV)’, was propagated in MARC-145 cells for seven passages. The PRRSV-1 field isolate AUT15-33 (GenBank Accession number MT000052), further referred to as the ‘high virulent strain (HV)’, was propagated for three passages in porcine alveolar macrophages as described before (9). Titers were determined on the respective cell line (MARC-145, MA-104 derived African Green monkey kidney cell line) or cells (porcine alveolar macrophages, PAMs) used for propagation.

### 2.3 Euthanasia and sample collection

Approximately 21 dpi (21 ± 2, gestation day 105 ± 2), gilts and their litters were anesthetized by intravenous injection of Ketamine (Narketan^®^ 100 mg/mL, Vetoquinol Österreich GmbH, Vienna Austria, 10 mg/kg body weight) and Azaperone (Stresnil^®^ 40 mg/mL, Elanco GmbH, Cuxhaven, Germany, 1.5 mg/kg body weight) and subsequently euthanized *via* intracardial injection of T61^®^ (Intervet GesmbH, Vienna, Austria, 1 mL/10 kg body weight). To retrieve samples, the abdomen of the gilts was opened, and the uteri removed, placed into a trough, and rinsed with tap water to remove maternal blood. The uteri were incised and opened at the anti-mesometrial side. The position of each fetus, from the left and right uterine horn, was recorded as previously described (38, 40). Fetal preservation status for each individual fetus was assessed and categorized as viable (VIA), meconium-stained (MEC), decomposed (DEC), and autolyzed (AUT) as previously described (39). For investigations on immune cell populations at the maternal-fetal interface, two fetuses per gilt were randomly selected and removed with their umbilical cord, placenta, and a portion of the uterus adjacent to the umbilical stump. A 1 × 1 cm piece of the maternal-fetal interface, was embedded in Tissue-Tek^®^ O.C.T compound (Sakura Fintek, Alphen aan den Rijn, The Netherlands) and immediately frozen in liquid isopentane whilst placed on dry ice and stored at –80 °C until further processing. The myometrium was trimmed off and the maternal endometrium (ME) and fetal placenta (FP) were mechanically separated with two forceps without contaminating either side. Once separated, 40 g of ME and 60 g of FP were collected in sterile collection cups (Greiner Bio-One, Frickenhausen, Germany) filled with medium (RPMI-1640 with stable L-glutamine supplemented with 100 IU/mL penicillin and 0.1 mg/mL streptomycin (PAN-Biotech, Aidenbach, Germany)). In addition, tissue pieces from the ME and FP for viral load quantification were snap-frozen in liquid nitrogen and stored at –80 °C until further processing.

### 2.4 Cell isolation

The procedure for the isolation of immune cells from the porcine maternal-fetal interface has been described previously (37). In brief, ME and FP tissues were cut into small pieces and incubated in tissue digestion medium [RPMI-1640 supplemented with 2% (v/v) heat-inactivated fetal calf serum (FCS; Sigma-Aldrich, Schnelldorf, Germany), 25 U/mL DNase type I (Thermo Fischer Scientific), 300 U/mL Collagenase type I (Thermo Fisher Scientific), 100 IU/mL penicillin (PAN-Biotech), and 0.1 mg/mL streptomycin (PAN-Biotech)] for 1 h at 37 °C and constant mixing. Remaining larger pieces of tissue and dead cells were removed by draining the cell suspensions through a coarse-meshed sieve and subsequent filtering through a layer of cotton wool. Suspensions were centrifuged (350 *× g*, 10 minutes, 4°C), resuspended in 40% Percoll (13 mL, Thermo Fisher Scientific), underlaid with 70% Percoll (13 mL, Thermo Fisher Scientific), and subjected to density gradient centrifugation (920 *× g*, 30 minutes, room temperature). Isolated leukocytes were washed four times (phosphate-buffered saline (PBS, 2x), RPMI-1640 + 5% FCS (1x), and RPMI-1640 + 10% FCS (1x)) and immediately used for immune phenotyping.

### 2.5 Viral load quantification *via* RT-qPCR

The extraction of PRRSV RNA from the ME and FP, and quantification of the viral load in these tissues has been described elsewhere (38). Briefly, tissues were homogenized in lysis buffer (QIAzol^®^ lysis reagent, QIAGEN GmbH, Hilden, Germany) with three stainless steel beads using a TissueLyser II instrument (QIAGEN GmbH). The homogenates were centrifuged, chloroform was added, and the tubes were vigorously vortexed and subsequently spun (13 000 *× g*, 5 minutes) to ensure phase separation. The aqueous phase was collected, and viral RNA was obtained using the Cador Pathogen Kit (QIAGEN GmbH) in a QiaCubeHT device (QIAGEN GmbH) following the manufacturer’s instructions. An ORF7-specific reverse transcription quantitative polymerase chain reaction (RT-qPCR) for the LV and HV strain, primers and probes listed in **Table 2**, was performed using the Luna Onestep RT PCR kit (New England Biolabs GmbH, Frankfurt am Main, Germany). The viral load, expressed as genome equivalents (GE), was determined based on the serial dilution of SP6 transcripts, specific to the PRRSV-1 isolates that were cloned into a pGEM-T vector (pLS69, Promega GmbH, Walldorf, Germany) and amplified. The cloned product was digested with DNaseI (New England Biolabs GmbH) and viral SP6 RNA was purified with the RNeasy kit (QIAGEN GmbH). Hereafter, a Quantus fluorometer and RNA-specific fluorescent dye (Promega) were used to determine the RNA concentration. The RNA concentration was multiplied with Avogadro’s number and divided by the molecular mass of the PRRSV-1 specific SP6 transcripts to determine the absolute quantity of GE.

**Table 2:** Overview primers and probes used for PRRSV ORF7-specific RT-qPCR.

	Forward primer (5’-3’)	Reverse primer (5’-3’)	Probe
LV	TCAACTGTGCCAGTTGCTGG	TGCGGCTTCTCAGGCTTTTTC	5′Fam-CCCAGCGCCAGCAAYCTAGGG Tamra-3′
HV	TCAACTGTGCCAGTTGCTGG	TGRGGCTTCTCAGGCTTTTC	5′Fam-CCCAGCGYCRRCARCCTAGGG Tamra-3′

### 2.6 Flow cytometry staining and analysis

Mononuclear immune cells (1.5 × 10^6^ cells per isolation) from ME and FP, were transferred into a 96-well round bottom microtiter plate (Greiner Bio-One) and stained in a 5- or 6-step procedure. An overview of the primary monoclonal antibodies (mAbs) and secondary reagents used per panel is given in **Table 3**. All incubation steps (20 minutes, 4°C) were followed by two washes with cold PBS supplemented with 10% (v/v) porcine plasma (in-house preparation) or as specified. Surface antigens were stained with mAbs listed in **Table 3** followed by incubation with secondary reagents. Free antibody sites of the isotype-specific secondary antibodies were blocked with 2 µg whole mouse IgG (ChromPure, Jackson ImmunoResearch, West Grove, PA, United States) and subsequently washed with PBS. Thereafter, a mixture of directly conjugated primary mAbs, streptavidin conjugates, and the Fixable Viability Dye eFluor 780 (Thermo Fisher Scientific) was applied. The BD Cytofix/Cytoperm kit (BD Biosciences, San Jose, CA, USA) was used to fix and permeabilize the cells. This was followed by a staining for intracellular antigens using directly conjugated mAbs. All samples were measured on a FACSCanto II flow cytometer (BD Biosciences) equipped with three lasers (405, 488, and 633 nm), and a minimum of 1 × 10^5^ lymphocytes per sample were recorded. Single-stained samples were prepared and recorded for automatic calculation of compensation, using FACSDiva software version 6.1.3 (BD Biosciences). The obtained data was analyzed with FlowJo software version 10.8.1 (BD Biosciences) and a consecutive gating strategy was applied (**Supplementary Figure 1**). A time gate was applied and based on the light scatter properties [forward scatter area (FSC-A) vs. side scatter area (SSC-A)] lymphocytes were identified. A 2-step doublet discrimination was performed and subsequently cells with high auto fluorescent signal were excluded using a 530/30 nm bandpass filter in the excitation line of the violet laser. Dead cells were excluded by a high signal for the Fixable Viability dye eFluor 780.

**Table 3:** Antibodies and secondary reagents used for FCM staining.

Antigen	Clone	Isotype	Source	Labeling	Fluorophore	
**Total mononuclear immune cells**						
CD45	K252.1E4	IgG1	Bio-Rad	Direct	AlexaFluor647	
**Myeloid cells**						
CD169	3B11/11	IgG1	Bio-Rad	Indirect^A^	AlexaFluor647	
CD14	Tük4	IgG2a	Bio-Rad	Indirect^B^	PE-Cy7	
CD163	2A10/11	IgG1	Bio-Rad	Direct	PE	
CD172a	74-22-15A	IgG2b	In-house	Indirect^C,D^	BV421	
**NK cells**						
CD3	BB23-8E6-8C8	IgG2a	BD biosciences	Direct	PerCP-Cy5.5	
CD8α	11/295/33	IgG2a	In-house	Indirect^D^	BV421	
CD172a	74-22-15	IgG1	In-house	Indirect^E^	PE	
NKp46	VIV-KM1	IgG1	In-house	Direct	AlexaFluor647	
CD16	G7	IgG1	Bio-Rad	Direct	FITC	
**B and T cells**						
CD4	74-12-4	IgG2b	BD biosciences	Direct	PerCP-Cy5.5	
CD8α	76-2-11	IgG2a	In-house	Indirect^B^	PE-Cy7	
CD27	b30c7	IgG1	In-house	Direct	AlexaFluor647	
CD79αcγ	HM57	IgG1	Thermo Fisher Scientific	Direct	PE	
TCR-γδ	PPT16	IgG2b	In-house	Indirect^F^	AlexaFluor488	
CD8β	PPT23	IgG1	In-house	Indirect^D^	BV421	

^A^Goat-anti-mouse anti-IgG1-AlexaFluor647, Thermo Fisher Scientific, ^B^Goat-anti-mouse anti-IgG2a-PE-Cy7, Southern Biotech, ^C^Goat-anti-mouse anti-IgG2b-biotin, Southern Biotech, ^D^Streptavidin-BV421, Biolegend, ^E^Goat-anti-mouse anti-IgG1-PE, Southern Biotech, ^F^Goat-anti-mouse anti-IgG2b-AlexaFluor488, Jackson Immuno Research.

### 2.7 Immunofluorescence histology staining

Tissue from the maternal-fetal interface was sectioned using a Leica CM1950 microtome (Leica Biosystems Nussloch GmbH, Nussloch, Germany). Sections were loaded onto a slide, air-dried at room temperature for 1 h, and fixed with methanol/acetone (1:1) for 30 minutes at -20°C. Slides were blocked with PBS + 5% goat serum (Vector Laboratories, Inc., Burlingame, CA, U.S.A.) for 30 minutes at room temperature. Mouse anti-PRRSV-NP mAb (IgG2a, clone P11/d72-c1, in-house, 1:2) was diluted in PBS and applied overnight (4°C). Thereafter, secondary goat anti-mouse IgG2a AlexaFluor488 (Thermo Fisher Scientific, 1:500) was diluted in PBS and applied for 40 minutes at room temperature. This was followed by a 2 h incubation with a rat anti-human/mouse Cytokeratin 8 mAb (1:500; IgG2a, clone TROMA-1, Merck KGaA, Darmstadt, Germany) and visualized by secondary goat-anti-rat IgG (H+L) AlexaFluor647 (1:500; Thermo Fisher Scientific), for 40 minutes, both at room temperature. After each incubation step, slides were washed three times in PBS for five minutes. Nuclei were stained with DAPI (Sigma Aldrich) for 3 minutes in the dark and the slides were washed twice with PBS. Finally, slides were washed once with dH_2_O and covered with mounting medium (Mowiol^®^4-88, Polysciences Europe GmbH, Germany) and a cover glass. Tissue sections were scanned using an Axioimager Z.1 microscope (Carl Zeiss Micro imaging GmbH, Germany) equipped with TissueFAXS hardware and software (TissueGnostics GmbH, Austria).

### 2.8 Statistics and graphical representation

The frequencies of major immune cells lineages (NK, γδ, B, CD4 T, and CD8β T cells), as a measure within viable lymphocytes, were exported into Microsoft Excel (Office 2016, Microsoft, Redmond, WA, United States) and corrected for CD45 expression as previously described (37). Also, frequencies of immune cell subsets and myeloid phenotypes were exported into Microsoft Excel and imported into GraphPad Prism version 9.2.0 (GraphPad Software Inc., San Diego, CA, United States) for the graphical presentation highlighting animal-to-animal variation. Statistical analysis was performed with R version *R v4.0.2* (41).

#### 2.8.1 Viral load quantification *via* RT-qPCR

We analyzed log_10_ transformed RT-qPCR measured viral loads, after adding a constant of one to every observation in the ME and FP tissue, *via* two separate univariate linear mixed effects models applying function *lmer* in R package *lme4 v1.1-27.1* (42) fitting a fixed categorical effect of treatment with the four factor levels involving a challenge: No.Vac_Chall_LV, Vac_Chall_LV, No.Vac_Chall_HV, and Vac_Chall_HV, respectively. We further included a random intercept for gilt with 16 factor levels (four gilts in each of the four treatment groups) as we had measures from two fetuses per gilt. Option *REML* was set to false to request maximum likelihood estimation. We then calculated estimated marginal means for each challenge group using function *emmeans* in package *emmeans v1.7.*5 Lenth (43) and requested hypothesis testing for all pairwise contrasts between estimated marginal means of treatment levels using option *pairwise*. Default multiple testing correction for these pairwise contrasts was turned off (option *adjust = “none”*). We performed a False Discovery Rate (FDR) multiple testing correction (44) across all p-values for all pairwise treatment contrasts across the two analyzed tissues. The multiple testing load was 12 tests total (six group comparisons × two tissues) and significance was declared at 10% FDR.

Results of the models are visualized *via* bar plots of estimated marginal means on a log_10_ transformed level using packages *RColorBrewer v1.1-2* (45), *ggplot2 v3.3.5* (46), and *ggpubr v0.4.0* (47) in which the fitted model is shown as the height of the bar plot. The black dots and whiskers represent upper and lower 95% confidence intervals of estimated marginal means. P-value brackets display contrasts significant at 10% FDR. Figures were exported as scalable vector graphics using package *svglite v2.0.0* (48).

#### 2.8.2 Viral load and CD163^high^CD169^pos^ phenotypes

To investigate the relationships between viral loads and CD163^high^CD169^pos^ phenotypes in both the ME and FP tissue, we produced scatterplots and calculated Spearman correlation coefficients on log_10_ transformed viral loads and log_10_ transformed CD163^high^CD169^pos^ phenotypes separately for each challenged group (No.Vac_Chall_LV, Vac_Chall_LV, No.Vac_Chall_HV, and Vac_Chall_HV), after adding a constant of 1 to every observation for both the viral load and cell type data. P-values in these plots were not corrected for multiple testing. Plots were produced using packages *RColorBrewer v1.1-2* (45), *ggplot2 v3.3.5* (46), and exported in svg format using package *svglite v2.0.0* (48).

#### 2.8.3 Immune cells

Our data comprised two different types of measurements. Frequencies of major immune cell lineages (*e.g.* total NK, total γδ T, total B, total CD4, and total CD8β T cells), phenotypes of the myeloid lineage (*e.g.* CD14^pos^CD172a^neg^, CD14^pos^CD163^high^CD169^pos^, and CD14^neg^CD163^high^CD169^pos^ cells), which were investigated in separate samples (**Table 3**) and frequencies of immune cell subsets as compositional data, derived from a single sample. Compositional data (CoDa) were transformed into log-ratios, to get rid of the constant sum constraint, allowing standard uni- and multivariate model employment for hypothesis testing (49).

For compositions of two components, which are perfectly negatively correlated (correlation coefficient of –1), and with components of the same effect sizes but with opposing signs (in our study CD8α^neg/dim^ *vs.* CD8α^high^ γδ T cells and CD14^pos^CD172a^pos^ *vs*. CD14^neg^CD172a^pos^ cells), we chose the former in each composition due to its higher discriminative power after log_10_ transformation during hypothesis testing. Immune cell subsets representing compositions of three components included NK cells (*i.e.* NKp46-defined subsets: NKp46^neg^, NKp46^pos^, and NKp46^high^), CD4 T cells (*i.e*. CD8α/CD27-defined subsets: CD8α^neg^CD27^pos^, CD8α^pos^CD27^pos^, and CD8α^pos^CD27^neg^), and CD8 T cells (*i.e*. CD8α/CD27-defined subsets: CD8α^pos^CD27^high^, CD8α^pos^CD27^pos^, and CD8α^pos^CD27^neg^). Each composition was subjected to centered log ratio (clr) transformation using function *clr* in package *compositions v2.0-4* (50, 51) after turning them into a package specific class of type *Aitchison compositions* using function *acomp*. Clr transformed data was then reformatted into “long data format” applying functions from package *dplyr v1.0.7* (52).

We then analyzed every measured immune cell type individually, either log_10_ transformed after adding a constant of one to every observation or clr transformed for the compositional data, fitting univariate linear mixed models applying function *lmer* in R package *lme4 v1.1-27.1* (42) changing the optimizer to *“nloptwrap”* with 100,000 iterations and setting option *REML* to false to perform maximum likelihood estimation to yield the most accurate estimates for the fixed effects part of the model. The fixed effects part of our models contained a main effect of treatment with six factor levels (No.Vac_No.Chall, Vac_No.Chall, No.Vac_Chall_LV, Vac_Chall_LV, No.Vac_Chall_HV, Vac_Chall_HV), a fixed effect of tissue type with levels ME and FP, and the interaction between treatment and tissue type. We further fitted a random intercept effect of day of experiment (six levels) to reduce any potential technical noise in our data. A random intercept of gilt (24 levels) was added to account for the covariance structure in our data (each gilt had measures of two fetuses each measured in the two tissues). As each level of random intercept of gilt had two observations per tissue, we added a dummy coded, centered, random slope for tissue as recommended by Barr et al. (53). Variance homogeneity of the residuals, normal distribution of residuals, fitted random intercepts, and slopes were verified with custom R scripts.

We then calculated estimated marginal means for all treatment levels for both tissues and tested for all pairwise differences (option *pairwise~treatment|tissue*) between treatment levels within tissue with function *emmeans* in package *emmeansv1.7.*5 (43). Default multiple testing correction for these pairwise contrasts was turned off (option *adjust = “none”*). We then selected pairwise biological contrasts of interest, excluding the contrasts “Vac_Chall_LV *vs.* No.Vac_Chall_HV” and “No.Vac_Chall_LV *vs.* Vac_Chall_HV”, and collected all p-values for all contrasts of interest, measured in all cell types for both tissues before applying a False Discovery Rate (FDR) multiple testing correction (44). Multiple testing correction was performed across all major immune cell lineages, myeloid phenotypes, and separately across all immune cell subsets. The multiple testing load was 234 tests total (13 group comparisons × nine phenotypes × two tissues). Significance was declared at 10% FDR. Modelling results were visualized with bar plots as described for viral load (in section 2.8.1).

#### 2.8.4 Graphical representation

All figures were assembled using Inkscape software version 1.1.1 (URL https://inkscape.org/)

## 3 Results

### 3.1 Viral load at the maternal-fetal interface and fetal preservation

**Figure 1:**
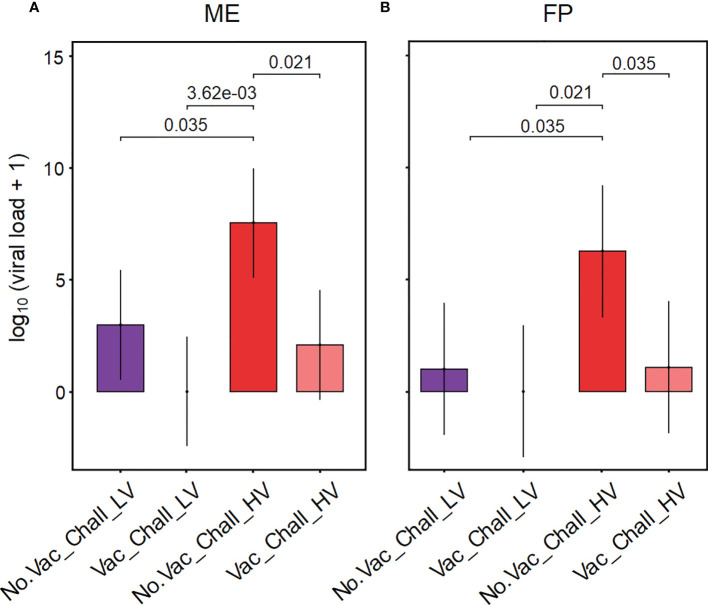
Viral RNA at the maternal-fetal interface of infected and vaccinated-infected gilts. Porcine reproductive and respiratory syndrome virus (PRRSV) RNA was extracted from tissue from the maternal endometrium (ME) and fetal placenta (FP). The viral load within the respective tissues was determined using an ORF7 PRRSV-1 field isolate specific RT-qPCR. PRRSV RNA was not detected in tissue samples from the No.Vac_No.Chall and Vac_No.Chall group and are therefore not shown. A linear mixed effects model fitting a fixed categorical effect (treatment) and random intercept for gilt (16 levels) was applied for the ME and FP separately. Results for the viral load are summarized in bar plots for the ME **(A)** and FP **(B)**. The y-axes show the estimated marginal means (emmeans) of the viral load (genome equivalents/g tissue) on a log_10_ scale, after adding a constant of + 1, for the four different treatment groups. Only significant p-values (p < 0.1) corrected for multiple testing, using a false discovery rate approach, across all pairwise comparisons of contrasts, across both tissues, are shown above the brackets. The whiskers depict the 95% confidence intervals of the emmeans. Depicted treatment groups: No.Vac_Chall_LV (dark purple, non-vaccinated and infected low virulent strain), Vac_Chall_LV (light purple, vaccinated and infected low virulent strain), No.Vac_Chall_HV (dark red, non-vaccinated and infected high virulent strain), and Vac_Chall_HV (light red, vaccinated and infected high virulent strain).

The viral load in tissues from the maternal-fetal interface was determined using RT-qPCR for PRRSV ORF7, with primers specific for the LV or the HV strain. Since no viral RNA for any strain could be detected in the ME and FP from gilts in the No.Vac_No.Chall and the Vac_No.Chall group (data not shown) only the challenged groups (No.Vac_Chall_LV, Vac_Chall_LV, No.Vac_Chall_HV, and Vac_Chall_HV) are displayed in **Figure 1**. Our analysis revealed that the emmeans for the viral load were significantly higher for the No.Vac_Chall_HV group as compared to the No.Vac_Chall_LV, Vac_Chall_LV, and Vac_Chall_HV group within ME and FP, respectively (**Figures 1A, B**). Of note, at the maternal-fetal interface from fetuses originating from Vac_Chall_LV gilts no viral RNA could be detected (**Supplementary Figure 2**). For Vac_Chall_HV gilts, viral RNA could be detected in the ME from a few fetuses from two different litters (gilts 15 and 16) and for gilt 15 in affected fetuses the virus was transmitted to the FP (**Supplementary Figure 2**), highlighting vertical transmission. Furthermore, there was a substantial negative impact on the fetal preservation status in the No.Vac_Chall_HV gilts (**Supplementary Figure 2**). Only 56% of these fetuses were designated as viable whereas in the other groups the vast majority (>90%) of fetuses were viable (data for the No.Vac_No.Chall and Vac_No.Chall group not shown). A clear difference in impact on the fetal preservation status between the two PRRSV-1 field isolates (LV and HV) was observed and demonstrated a divergence in virulence.

### 3.2 CD172a^pos^ cells at the maternal-fetal interface and their correlation with viral load

**Figure 2:**
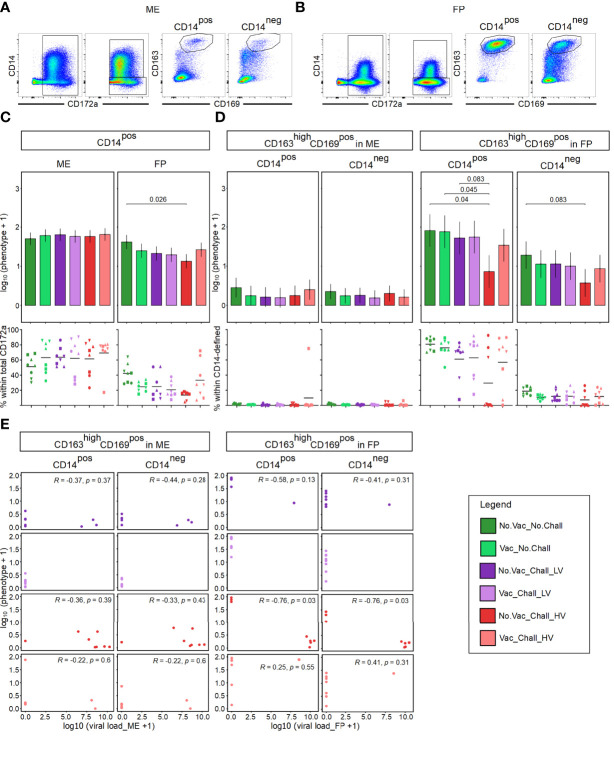
Mononuclear phagocytes at the maternal-fetal interface and their correlation to viral load. **(A, B)** A time gate was applied and mononuclear phagocytes (MPCs) were gated based on their SSC-A (side scatter area) versus FSC-A (forward scatter area) characteristics and following a consecutive gating strategy was applied to exclude doublets, cells with high autofluorescence, and dead cells (Supplementary Figure 1). MPCs were further analyzed for their expression of CD172a and subsequently sub-gated for CD14^pos^CD172a^pos^ and CD14^neg^CD172a^pos^ MPCs. The two CD14-defined MPC populations were further analyzed for their co-expression of CD163 and CD169. For both CD14-defined MPCs a CD163^high^CD169^pos^ subset was identified at the maternal fetal interface. Representative pseudocolor plots from the maternal endometrium (ME) in **(A)** and fetal placenta (FP) in **(B)** from a No.Vac_No.Chall fetus are shown. **(C)** A linear mixed effects model considering the fixed effects of treatment, tissue, and the interaction between both was applied. A random intercept (gilt) was fitted and estimated marginal means (emmeans) were calculated. Results for total CD14^pos^ MPCs within the ME (left) and FP (right) are presented as bar plots on top. On the y-axes the estimated marginal means (emmeans) for CD14^pos^ MPCs on a log_10_ scale, after adding a constant of + 1, are depicted. The graphs below depict the frequency of CD14^pos^ MPCs within total CD172a^pos^ cells for the individual fetuses within each treatment group in the ME (left) and FP (right). **(D)** A linear mixed effects model considering the fixed effects of treatment, tissue, and the interaction between both was applied. A random intercept (gilt) was fitted and estimated marginal means (emmeans) were calculated. Results for CD163^high^CD169^pos^ MPCs within CD14^pos^ and CD14^neg^ cells within the ME (left) and FP (right) are shown. The y-axes in the bar plots (on top) represent the emmeans of the CD163^high^CD169^pos^ MPCs within CD14-defined subsets on a log_10_ + 1 scale. The frequencies of the CD163^high^CD169^pos^ MPCs within CD14-defined subsets for the individual fetuses and anatomic locations are given in the graphs below. For all bar plots only significant p-values (p < 0.1), corrected for multiple testing using a false discovery rate approach across all 234 pairwise comparisons of contrasts, are shown above the brackets. The whiskers depict the 95% confidence intervals of the emmeans. For all graphs showing the frequencies of a specific cell subset, results for the fetuses from one gilt are represented by different symbols. The black bars in the graphs display the mean within the respective treatment group within the specified anatomic location. **(E)** Spearman correlation coefficients were estimated, to investigate the relationship between log_10_ transformed CD163^high^CD169^pos^ CD14-defined MPCs and log_10_ transformed viral load, for all challenged groups and both anatomic locations. Results for the spearman correlation in the ME are shown on the left and FP on the right. The correlation coefficients **(R)** and p-values (p < 0.1) not corrected for multiple testing are depicted. For all bar plots, graphs, and scatterplots the depicted treatment groups are: No.Vac_No.Chall (dark green, non-vaccinated and non-infected), Vac_No.Chall (light green, vaccinated and non-infected), No.Vac_Chall_LV (dark purple, non-vaccinated and infected low virulent strain), Vac_Chall_LV (light purple, vaccinated and infected low virulent strain), No.Vac_Chall_HV (dark red, non-vaccinated and infected high virulent strain), and Vac_Chall_HV (light red, vaccinated and infected high virulent strain).

Since PRRSV infects cells of the myeloid lineage, we sought to investigate their phenotype at the maternal-fetal interface. Following FCM staining, myeloid cells at the maternal-fetal interface were identified based on their CD172a expression and were subsequently divided into CD14^pos^ and CD14^neg^ subsets (**Figures 2A, B**). No significant differences were observed for the CD14^pos^ cells within total CD172a^pos^ cells in the ME whereas a significant decrease was observed in the FP of No.Vac_Chall_HV group as compared to No.Vac_No.Chall group (**Figure 2C**, top panel). In addition, a high degree of variation between individual fetuses, especially within the ME, was identified (**Figure 2C**, bottom panel, scatterplots). Both CD14-defined subsets were further analyzed for their co-expression of CD163 and CD169, both molecules involved in viral entry, and CD163^high^CD169^pos^ mononuclear phagocytes (MPCs) were identified (**Figures 2A, B**). The abundance of these CD163^high^CD169^pos^ MPC phenotypes in the respective CD14-defined populations in ME was rather low (**Figures 2A, D** left) and no differences in emmeans for either macrophage phenotypes was observed between the six groups (**Figure 2D** left, top panel). A high abundance of CD163^high^CD169^pos^ phenotypes within CD14^pos^ and CD14^neg^ CD172a^pos^ cells was found in the FP, especially in the No.Vac_No.Chall and the Vac_No.Chall groups (**Figures 2B, D** right, bottom panel). A significant drop for CD163^high^CD169^pos^ cells within CD14^pos^ CD172a^pos^ MPCs was seen in the FP of fetuses from the No.Vac_Chall_HV as compared to the No.Vac_No.Chall, Vac_No.Chall, and No.Vac_Chall_LV groups (**Figures 2B, D** right, top panel). For CD163^high^CD169^pos^ cells within CD14^neg^ MPCs in the FP a similar drop was observed for the No.Vac_Chall_HV as compared to the No.Vac_No.Chall group (**Figures 2B, D** right, top panel). These significant contrasts for both MPC subsets in the FP (**Figure 2D** right) prompted us to investigate a correlation with viral load. For this purpose, a spearman correlation was performed for all challenged groups and both anatomic locations (**Figure 2E**). A strong negative correlation (R = –0.76, p = 0.03) between both MPC phenotypes and the viral load was revealed in the FP from No.Vac_Chall_HV fetuses. Furthermore, virus infected cells, as identified with a monoclonal antibody targeting PRRSV-NP, were predominantly detected in the FP (**Supplementary Figure 3**).

### 3.3 Major lymphocyte subsets at the maternal-fetal interface in response to an infection with PRRSV

**Figure 3:**
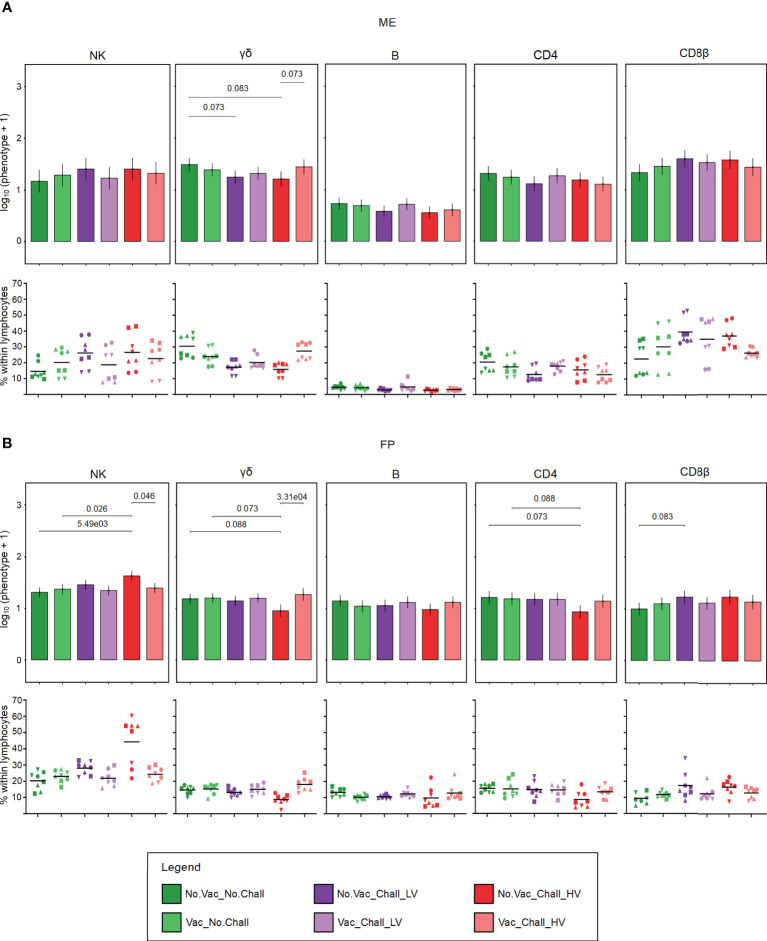
Major lymphocyte subsets at the maternal-fetal interface. Following the applied consecutive gating strategy (**Supplementary Figure 1**) NK cells, γδ T cells, B cells, CD4 T cells, and CD8β T cells were identified in the ME **(A)** and FP **(B)**. A linear mixed effects model considering the fixed effects of treatment, tissue, and the interaction between both was applied. A random intercept (gilt) was fitted and estimated marginal means (emmeans) were calculated. The bar plots (top panel; **(A)** ME; **(B)** FP) depict the results for the obtained major lymphocyte subsets across all treatment groups and are presented as emmeans of each subset on a log_10_ + 1 scale as depicted on the y-axes. Only significant p-values (p < 0.1), corrected for multiple testing using a false discovery rate approach, across all 234 pairwise comparisons of contrasts, are shown above the brackets. The whiskers depict the 95% confidence intervals of the emmeans. Frequencies of the major lymphocyte subsets, within viable lymphocytes corrected for CD45 expression, are given (bottom panel; **(A)** ME; **(B)** FP). For all graphs, results for each individual fetus are shown and different symbols indicate fetuses from different gilts. The black bars in the graphs display the mean within the respective treatment group within the specified anatomic location. For all bar plots and graphs shown, the depicted treatment groups are: No.Vac_No.Chall (dark green, non-vaccinated and non-infected), Vac_No.Chall (light green, vaccinated and non-infected), No.Vac_Chall_LV (dark purple, non-vaccinated and infected low virulent strain), Vac_Chall_LV (light purple, vaccinated and infected low virulent strain), No.Vac_Chall_HV (dark red, non-vaccinated and infected high virulent strain), and Vac_Chall_HV (light red, vaccinated and infected high virulent strain).

Next to MPCs, major lymphocyte subsets were investigated by flow cytometry and the applied gating strategy is illustrated in **Supplementary Figure 1**. A CD3^neg^CD8α^pos^CD16^pos^CD172a^neg^ phenotype was used to identify NK cells. During steady state conditions (No.Vac_No.Chall), total NK cells were present in similar frequencies within total lymphocytes in both the ME and FP (**Figure 3**, Scatter plots). With regards to PRRSV-mediated changes, no significant contrasts were detected in the ME, possibly due to the high degree of animal-to-animal variation. Significant higher emmeans for total NK cells could be observed in the FP from No.Vac_Chall_HV fetuses. Significant contrasts for the FP were found between No.Vac_Chall_HV vs No.Vac_No.Chall, No.Vac_Chall_HV vs Vac_No.Chall, and No.Vac_Chall_HV vs Vac_Chall_HV (**Figure 3B**). Porcine γδ T cells at the maternal-fetal interface were identified with a monoclonal antibody targeting a T-cell receptor γδ-specific CD3ϵ chain (clone PPT16) (54). Emmeans for the total γδ T cells in the ME were lower for both non-vaccinated challenged groups (No.Vac_Chall_LV and No.Vac_Chall_HV) as compared to the No.Vac_No.Chall group. Furthermore, the vaccination seemed to have prevented this loss in total γδ T cells in the Vac_Chall_HV group. These differences in emmeans were also visible in the scatterplots showing the percentages of γδ T cells within lymphocytes (**Figure 3A**). Total γδ T cells were significantly reduced in the FP of fetuses from the No.Vac_Chall_HV group as compared to No.Vac_No.Chall, Vac_No.Chall, and vaccinated counterpart (Vac_Chall_HV) (**Figure 3B**). Total B cells at the maternal-fetal interface were identified using the pan-B cell marker CD79α. For this phenotype, no PRRSV-associated changes were observed neither in the ME nor in the FP (**Figure 3**). CD4 and CD8 T cells, were characterized by gating on total CD4 and total CD8β expressing T cells (**Supplementary Figure 1**). No significant PRRSV-induced contrasts for both T cell phenotypes in the ME could be identified by our statistical model. However, for the total CD8β T cells a high degree of animal-to-animal variation was observed for most groups except for the Vac_Chall_HV group. A significant reduction in total CD4 T cells could be observed in the FP from the No.Vac_Chall_HV group as compared to the No.Vac_No.Chall and Vac_No.Chall groups. For CD8β T cells, we only observed a significant increase in the No.Vac_Chall_LV group in comparison to the No.Vac_No.Chall group (**Figure 3B**).

### 3.4 NKp46-defined NK cell phenotypes

**Figure 4:**
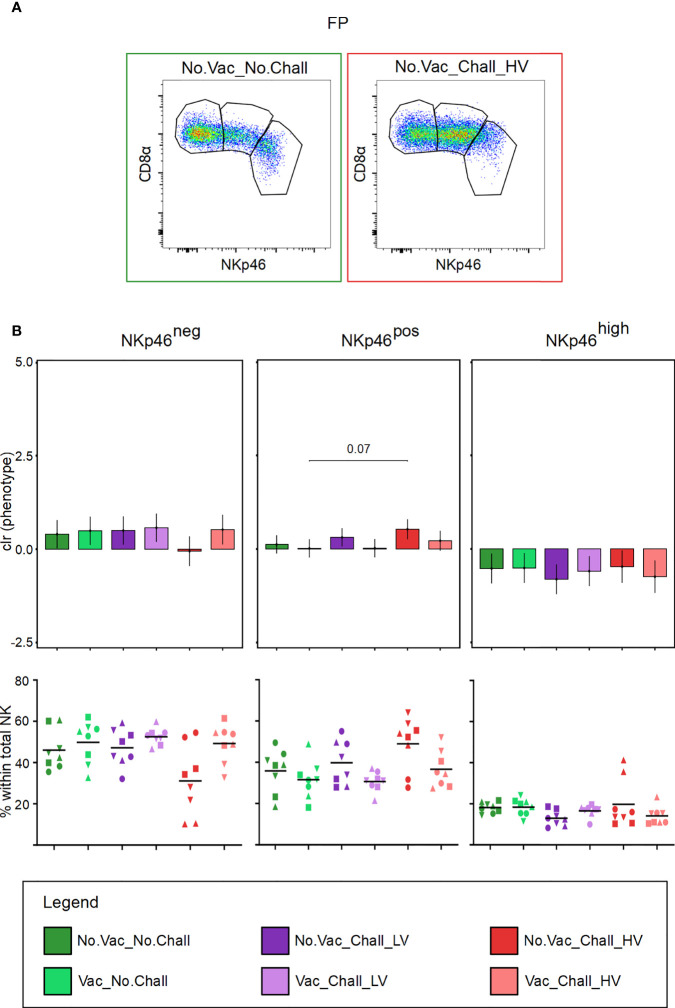
NKp46-defined NK cell subsets in the fetal placenta. **(A)** CD3^neg^CD8α^pos^CD16^pos^CD172a^neg^ NK cells in the fetal placenta (FP) were investigated for their expression of NKp46. Three NK cell subsets were identified: NKp46^neg^, NKp46^pos^, and NKp46^high^ (from left to right). Representative pseudocolor plots for the FP from a No.Vac_No.Chall and No.Vac_Chall_HV fetus are shown. **(B)** Univariate compositional data analysis was performed for the three NKp46-defined NK cell subsets. Results are represented in the bar charts (top panel). The y-axes depict the estimated marginal means (emmeans) of the centered log ratios (clr) transformed NKp46^neg^, NKp46^pos^, and NKp46^high^ NK cell subsets (from left to right). Only significant p-values (p < 0.1), corrected for multiple testing using a false discovery rate approach, across all pairwise comparisons of contrasts for all (nine) compositional cell subsets and both tissues, are shown above the brackets. The whiskers depict the 95% confidence intervals of the clr-transformed data. The graphs in the bottom panel show the frequencies of the three NKp46-defined subsets within total NK cells. For all graphs, results for each individual fetus are shown and different symbols indicate fetuses from different gilts. The black bars in the graphs display the mean within the respective treatment group. For all bar plots and graphs shown, the depicted treatment groups are: No.Vac_No.Chall (dark green, non-vaccinated and non-infected), Vac_No.Chall (light green, vaccinated and non-infected), No.Vac_Chall_LV (dark purple, non-vaccinated and infected low virulent strain), Vac_Chall_LV (light purple, vaccinated and infected low virulent strain), No.Vac_Chall_HV (dark red, non-vaccinated and infected high virulent strain), and Vac_Chall_HV (light red, vaccinated and infected high virulent strain).

Total CD3^neg^CD8α^pos^CD16^pos^CD172a^neg^ NK cells in the FP were further investigated for their expression of NKp46. Three NK cell subsets, NKp46^neg^, NKp46^pos^, and NKp46^high^ were identified. Representative pseudocolor plots for one fetus from the No.Vac_No.Chall and No.Vac_Chall_HV group are shown in **Figure 4A**. Considering that the relative frequencies of the three NKp46-defined NK cell subsets are interdependent, a univariate CoDa was performed. Therefore, to correct for this interdependence our data was transformed to centered log ratios (clr). The output of our model, showed a significant increase in NK cells with a NKp46^pos^ phenotype in the No.Vac_Chall_HV as compared to the Vac_No.Chall group (**Figure 4B**). For the other two NKp46-defined NK cell phenotypes, no significant changes were observed. When considering the raw frequency data, however, a visual reduction in the NKp46^neg^ NK cells in the FP from the No.Vac_Chall_HV group could be observed. Notably, considerable variation between individual fetuses was observed. Data on NKp46-defined NK cell phenotypes in the ME are not shown, since no significant changes were observed.

### 3.5 CD8α-defined γδ T cell phenotypes

**Figure 5:**
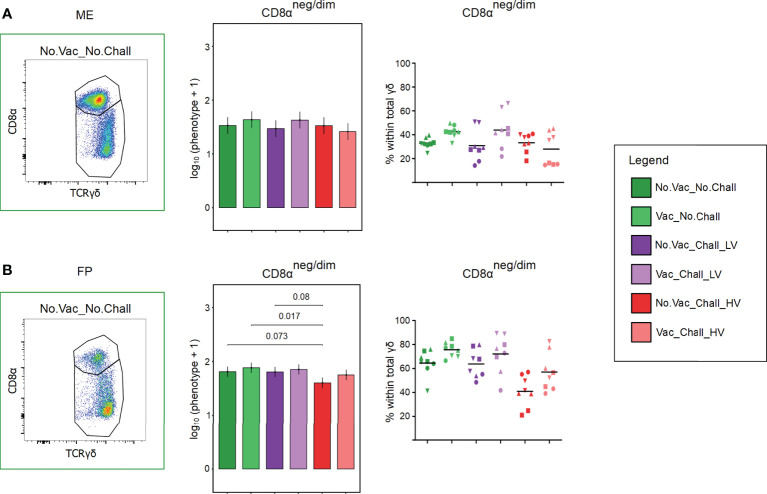
CD8α-defined γδ T cell phenotypes at the maternal-fetal interface. **(A, B)** Total γδ T cells were analyzed for their CD8α surface expression and separated into a CD8α^neg/dim^ and a CD8α^high^ γδ T cell subset. Representative pseudocolor plots for the **(A)** maternal endometrium (ME) and the **(B)** fetal placenta (FP) from a No.Vac_No.Chall fetus are given. A linear mixed effects model considering the fixed effects of treatment, tissue, and the interaction between both was applied. A random intercept (gilt) was fitted and estimated marginal means (emmeans) were calculated. The bar plots (middle panel; **(A)** ME; **(B)** FP), depict the results for the obtained CD8α^neg/dim^ γδ T phenotype across all treatment groups and are presented as emmeans of the CD8α^neg/dim^ γδ T cells on a log_10_ + 1 scale as depicted on the y-axes. Only significant p-values (p < 0.1), corrected for multiple testing using a false discovery rate approach, across all 234 pairwise comparisons of contrasts, are shown above the brackets. The whiskers depict the 95% confidence intervals of the emmeans. The frequencies of this CD8α^neg/dim^ phenotype within total γδ T cells are given in the graph (right panel; **(A)** ME; **(B)** FP). For all graphs, results for each individual fetus are given and different symbols indicate fetuses from different gilts. The black bars in the graphs display the mean within the respective treatment group within the specified anatomic location. For all bar plots and graphs shown, the depicted treatment groups are: No.Vac_No.Chall (dark green, non-vaccinated and non-infected), Vac_No.Chall (light green, vaccinated and non-infected), No.Vac_Chall_LV (dark purple, non-vaccinated and infected low virulent strain), Vac_Chall_LV (light purple, vaccinated and infected low virulent strain), No.Vac_Chall_HV (dark red, non-vaccinated and infected high virulent strain), and Vac_Chall_HV (light red, vaccinated and infected high virulent strain).

Total γδ T cells were analyzed for their expression of CD8α which enabled us to identify a CD8α^neg/dim^ and CD8α^high^ expressing subset in the ME and FP. Representative pseudocolor plots for the two investigated anatomic locations are shown in **Figures 5A, B**. CD8α^high^ expressing γδ T cells were the main phenotype in the ME (**Figure 5A**) whereas the CD8α^neg/dim^ expressing γδ T cells were more abundant in the FP (**Figure 5B**). For the statistical analysis, only γδ T cells with a CD8α^neg/dim^ phenotype were included since the effect size of the CD8α^high^ γδ T cells is dependent on the CD8α^neg/dim^ phenotype. In the ME no significant difference was found for the CD8α^neg/dim^ phenotype (**Figure 5A**). Nonetheless, a significant reduction for this phenotype and thus an increase in CD8α^high^ γδ T was observed in the FP of fetuses from the No.Vac_Chall_HV group as compared to the No.Vac_No.Chall, Vac_No.Chall, and No.Vac_Chall_LV group (**Figure 5B**).

### 3.6 The activation and differentiation state of porcine CD4 T cells

**Figure 6:**
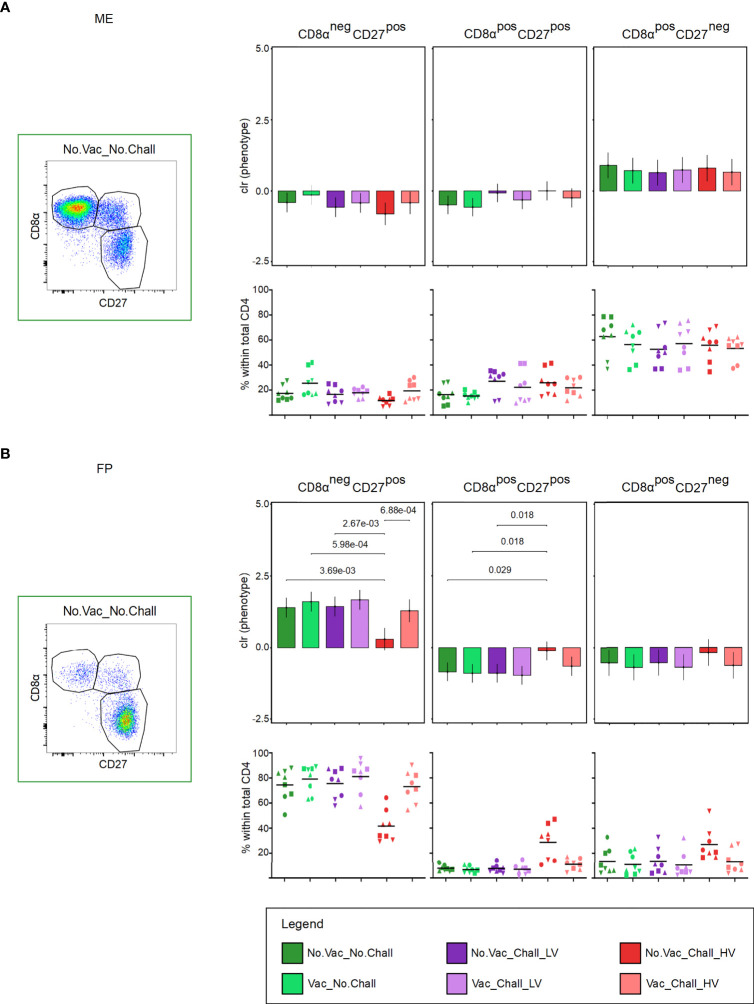
CD8α and CD27 expression of CD4 T cells at the maternal-fetal interface. **(A, B)** Total CD4 T cells were investigated for their expression of CD8α and CD27. CD8α^neg^CD27^pos^ (representing naive), CD8α^pos^CD27^pos^ (representing early effectors or central memory, Tcm), and CD8α^pos^CD27^neg^ (representing late effectors of effector memory, Tem) cells were identified. Representative pseudocolor plots for the **(A)** maternal endometrium (ME) and the **(B)** fetal placenta (FP) from a No.Vac_No.Chall fetus are shown. Univariate compositional data analysis was performed for the three CD8α/CD27-defined CD4 T cell subsets. Results are represented in the bar charts (top panel; **(A)** ME; **(B)** FP). The y-axes depict the estimated marginal means (emmeans) of the centered log ratios (clr) for the specified CD4 T cell subset. Only significant p-values (p < 0.1), corrected for multiple testing using a false discovery rate approach, across all pairwise comparisons of contrasts for all (nine) compositional cell subsets and both tissues, are shown above the brackets. The whiskers depict the 95% confidence intervals of the clr-transformed data. The graphs in the bottom panel **(A)** ME; **(B)** FP) show the frequencies of the CD8α/CD27-defined CD4 T cell subsets within total CD4 T cells. For all graphs, results for each individual fetus are shown and different symbols indicate fetuses from different gilts. The black bars in the graphs display the mean within the respective treatment group. For all bar plots and graphs shown, the depicted treatment groups are: No.Vac_No.Chall (dark green, non-vaccinated and non-infected), Vac_No.Chall (light green, vaccinated and non-infected), No.Vac_Chall_LV (dark purple, non-vaccinated and infected low virulent strain), Vac_Chall_LV (light purple, vaccinated and infected low virulent strain), No.Vac_Chall_HV (dark red, non-vaccinated and infected high virulent strain), and Vac_Chall_HV (light red, vaccinated and infected high virulent strain).

Total CD4 T cells at the maternal-fetal interface were investigated for their expression of CD8α and CD27 (**Figure 6**). This enabled us to delineate three subsets with a CD8α^neg^CD27^pos^ naive, CD8α^pos^CD27^pos^ early effector or central memory (Tcm), and CD8α^pos^CD27^neg^ late effector or effector memory phenotype (Tem) (**Figures 6A, B**), representative pseudocolor plots are shown). Since the three CD8α/CD27-defined CD4 T cell subsets are interdependent on each other, the components of the compositions were clr transformed before hypothesis testing to deal with the constant sum constraints. No significant changes in the CD8α/CD27-defined CD4 T cell subsets were observed in the ME (**Figure 6A**). In the FP, however, a significant decrease in CD8α^neg^CD27^pos^ naive CD4 T cells and a concurrent increase in CD8α^pos^CD27^pos^ Tcm cells was observed in the No.Vac_Chall_HV group as compared to the No.Vac_No.Chall, Vac_No.Chall, and No.Vac_Chall_LV group (**Figure 6B**). Of note, the five FP tissues with the highest CD8α^pos^CD27^pos^ percentages tested PRRSV positive in this tissue (fetuses G22 L7, G22 R10, G23 L5, G23 R11, and G24 L2, **Supplementary Figure 2**) and showed a reduced number of CD163^high^CD169^pos^ MPCs (**Figure 2D**). Furthermore, the significant loss in CD8α^neg^CD27^pos^ naive CD4 T cells was also observed as compared to the Vac_Chall_HV. However, in this case the increase in CD8α^pos^CD27^pos^ Tcm cells in the No.Vac_Chall_HV compared to the Vac_Chall_HV was not significant.

### 3.7 CD8β T cell phenotypes

**Figure 7:**
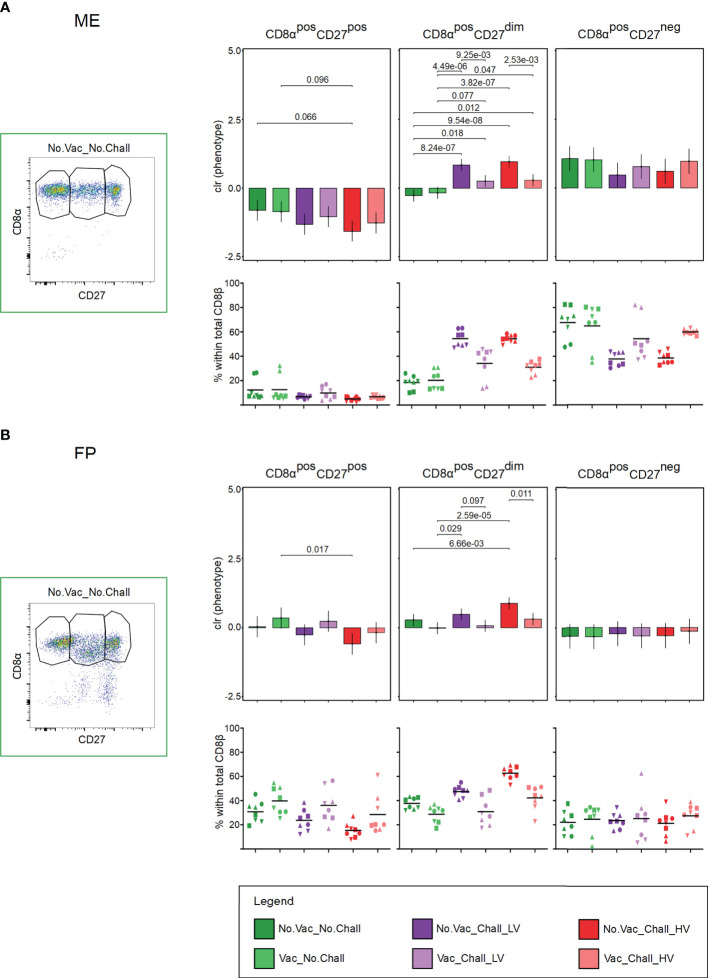
CD8α and CD27 expression of CD8β T cells at the maternal-fetal interface **(A, B)** Total CD8β T cells were investigated for their expression of CD8α and CD27. CD8β T cells with a CD8α^pos^CD27^pos^, CD8α^pos^CD27^dim^, and CD8α^pos^CD27^neg^ phenotype were identified and presumably represent naive, early effector, and late effector CD8 T cells, respectively. Representative pseudocolor plots for the **(A)** maternal endometrium (ME) and the **(B)** fetal placenta (FP) from a No.Vac_No.Chall fetus are shown. Representative pseudocolor plots for the **(A)** ME and the **(B)** FP from a No.Vac_No.Chall fetus are shown. Univariate compositional data analysis was performed for the three CD8α/CD27-defined CD8β T cell subsets. Results are represented in the bar charts (top panel; **(A)** ME; **(B)** FP). The y-axes depict the estimated marginal means (emmeans) of the centered log ratios (clr) transformed specified CD8β T cell subset. Only significant p-values (p < 0.1), corrected for multiple testing using a false discovery rate approach, across all pairwise comparisons of contrasts for all (nine) compositional cell subsets and both tissues, are shown above the brackets. The whiskers depict the 95% confidence intervals of the clr-transformed data. The graphs in the bottom panel **(A)** ME; **(B)** FP) show the frequencies of the CD8α/CD27-defined subsets within total CD8β T cells. For all graphs, results for each individual fetus are shown and different symbols indicate fetuses from different gilts. The black bars in the graphs display the mean within the respective treatment group. For all bar plots and graphs shown, the depicted treatment groups are: No.Vac_No.Chall (dark green, non-vaccinated and non-infected), Vac_No.Chall (light green, vaccinated and non-infected), No.Vac_Chall_LV (dark purple, non-vaccinated and infected low virulent strain), Vac_Chall_LV (light purple, vaccinated and infected low virulent strain), No.Vac_Chall_HV (dark red, non-vaccinated and infected high virulent strain), and Vac_Chall_HV (light red, vaccinated and infected high virulent strain).

As CD8 T cells are major effector cells in many viral infections, we sought to investigate their phenotype at the maternal-fetal interface. Therefore, the expression of CD8α and CD27 on the identified CD8β T cells was evaluated. CD8β T cells with a CD8α^pos^CD27^pos^, CD8α^pos^CD27^dim^, and CD8α^pos^CD27^neg^ phenotype were identified (**Figures 7A, B**, representative pseudocolor plots are shown) and represent CD8β T cells with a naive, early effector, and late effector phenotype, respectively (55, 56). The interdependency between the three CD8β T cell phenotypes was corrected for with CoDa. Several significant contrasts were identified in both investigated anatomic compartments. In the ME a significant loss of CD8β T cells with a CD8α^pos^CD27^pos^ naive phenotype and an accompanying increase of CD8α^pos^CD27^dim^ early effector phenotype was observed from No.Vac_Chall_HV fetuses as compared to the No.Vac_No.Chall and Vac_No.Chall group (**Figure 7A**). A similar increase of CD8α^pos^CD27^dim^ early effector CD8β T cells was observed for the ME from No.Vac_Chall_LV group as compared to the No.Vac_No.Chall and Vac_No.Chall groups (**Figure 7A**). In addition, significant contrasts for CD8β T cells with a CD8α^pos^CD27^dim^ early effector phenotype were observed between the non-vaccinated challenged groups, No.Vac_Chall_HV and No.Vac_Chall_LV, and their vaccinated counterparts, Vac_Chall_HV and Vac_Chall_LV, respectively (**Figure 7A**). Furthermore, a significant but limited increase in CD8β T cells with a CD8α^pos^CD27^dim^ early effector phenotype was observed in the Vac_Chall_HV and Vac_Chall_LV groups as compared to the No.Vac_No.Chall and Vac_No.Chall groups (**Figure 7A**). In the FP, a significant loss of CD8α^pos^CD27^pos^ naive CD8β T cells in the No.Vac_Chall_HV group as compared to the Vac_No.Chall group concurred with a strong increase in CD8β T cells with a CD8α^pos^CD27^dim^ early effector phenotype (**Figure 7B**). Also for No.Vac_Chall_LV group a significant increase of CD8β T cells with a CD8α^pos^CD27^dim^ early effector phenotype was observed (**Figure 7B**). Similarly to the ME, significant contrasts in the FP were observed between the non-vaccinated challenged groups, No.Vac_Chall_HV and No.Vac_Chall_LV, and their vaccinated counterparts, Vac_Chall_HV and Vac_Chall_LV, respectively (**Figure 7B**). Compared to the other investigated lymphocyte subsets, CD8α^pos^CD27^dim^ early effector CD8β T cells showed the strongest response to infection with the two PRRSV-1 strains.

## 4 Discussion and conclusions

Research on PRRSV-specific immune responses *in utero* is sparse. By using our previously established method of ME and FP separation (37), we were able to provide an in-depth characterization of the mononuclear immune cells at the maternal-fetal interface following experimental infection and vaccination.

In this study, two PRRSV-1 field isolates were used, designated in hindsight as LV and HV. Initially, we did not expect to see a difference in terms of reproductive failure, as both PRRSV-1 field isolates caused severe clinical signs in affected farms (9), as communicated by veterinarians in the field. However, viral loads measured in the ME and FP for the LV strain were significantly lower as compared to the HV strain for non-vaccinated animals **(** **Figure 1** **)**. Furthermore, for the LV infected gilts viral transmission from the ME to the FP was only observed in five fetuses. In addition, only two fetuses from LV infected gilts had an impaired fetal preservation status whereas in the HV infected gilts the fetal preservation was affected in many (n=30) fetuses (**Supplementary Figure 2**). An obvious explanation for these observed differences might be the *in vitro* passaging of the LV strain on MARC-145 cells (MA-104 derived African Green monkey kidney cell line), whereas the HV strain was passaged on porcine alveolar macrophages. It has been shown that PRRSV loses its virulence due to adaptation to MARC-145 cells *in vitro* resulting in an attenuated phenotype *in vivo* (57). Furthermore, PRRS MLVs can be generated by *in vitro* passaging leading to attenuation (58, 59). The LV strain has a 99.76% sequence homology to the PRRSV field isolate IVI-1173 (Genbank Accession number KX622783.1) that caused a PRRSV outbreak in Switzerland (2012) (60). Although not planned at the outset, these differences in virulence allowed valuable insights into the response of the investigated immune cell phenotypes, as outlined above, and discussed in the following.

In our reproductive gilt model, the PRRSV-1 based MLV (ReproCyc^®^ PRRS EU) completely or partially prevented reproductive signs following heterologous challenge with the LV PRRSV-1 field isolate and HV PRRSV-1 field isolate, respectively. Nevertheless, for gilts from the Vac_Chall_HV group viral transmission to the FP only occurred in one out of four litters. For the viral load, in the ME and FP, no significant difference could be found between the No.Vac_Chall_LV and the Vac_Chall_LV groups **(** **Figure 1** **)**. However, when considering the fetal preservation status and viral load of each given individual fetus it becomes apparent that no viral RNA could be detected at the maternal-fetal interface from Vac_Chall_LV gilts (**Supplementary Figure 2**). This is due to the fact that all observations for viral load in the Vac_Chall_LV gilts were zero resulting in the absence of variation in this group. In the Vac_Chall_HV group the viral load in the ME and FP was significantly lower as compared to the non-vaccinated counterpart. Furthermore, the fetal preservation status substantially improved when the gilts were vaccinated prior PRRSV infection **(** **Supplementary Figure 2** **)**.

We focused mainly on immune cell phenotypes *in utero*. Humoral-mediated effector mechanisms were not investigated but could also have contributed to the protective effects of the MLV. Following a similar vaccination scheme, PRRSV-specific antibodies were readily detected in the serum of vaccinated gilts after two MLV doses, which did not drastically change after a third dose (38). Combining the three dose MLV with the experimental infection with a PRRSV-1 field isolate significantly increased the antibody response in these gilts (38). In addition, serum transfer experiments in gestating females have shown that vertical transmission can be prevented by PRRSV-specific Nabs (61). Therefore, it is conceivable that PRRSV-specific antibodies, as detected in the serum, could be locally active *in utero* in the Vac_Chall_HV and Vac_Chall_LV group, and contribute to the protective effect of the vaccine.

As cells from the myeloid lineage are the primary targets for the virus; we characterized them using CD14, CD163, CD169 and CD172a. In the ME, CD172a^pos^ cells with a CD14^pos^ and CD14^neg^ phenotype were identified; however, the frequency of CD163^high^CD169^pos^ MPCs was rather low as compared to the FP **(** **Figures 2C, D** **)**. Similarly, other researchers evaluated the presence of CD163^pos^ and CD169^pos^ cells at the maternal-fetal interface and reported that they were significantly enriched in the FP during steady state and even 21 dpi with PRRSV-2 (31). In our study, the MPCs identified in the ME did not seem to be affected by the vaccination or infection since no significant differences were found. In contrast, in the ME of PRRSV-2 infected gilts an increase of CD163^pos^ and CD169^pos^ cells was found 21 dpi (31). In addition, another study demonstrated the increase in CD169^pos^ cells in both the ME and FP from PRRSV-1 inoculated sows as compared to controls at 10 dpi whereas the CD163^pos^ cell count was not altered (33). They also showed a decrease in CD14^pos^ cells in the FP of PRRSV-infected animals (33), this decrease is in line with the outcome of our study **(** **Figure 2D** **)**. Furthermore, we observed a significant loss in CD163^high^CD169^pos^ MPCs in the FP of the No.Vac_Chall_HV group, which was inversely associated to the viral load **(** **Figure 2E** **)**. The latter would be in line with the inverse relationship between placental CD163^pos^ cells and viral load in the fetal thymus (31). It has been shown that PRRSV induces apoptosis of PRRSV-infected cells, expressing CD163, and bystander apoptosis of virus-negative cells (34). Therefore, our data suggests that viral replication in the FP accounts for the observed loss of CD163^high^CD169^pos^ MPCs. The discrepancies observed as compared to the other studies, might be explained by the different methodologies used. So far, most investigations utilized immunofluorescence microscopy, which is limited in the number of cellular markers that can be investigated simultaneously. Flow cytometry enabled us to include multiple parameters for the characterization of the immune cells, although, at the cost of the spatial information in the tissue. Furthermore, our data indicates that there is a high degree of MPC heterogeneity at the maternal-fetal interface, which illustrates a need for more sophisticated phenotypical, transcriptional, and functional analyses in the context of PRRSV.

NK cells form a first line of defense in many viral infections (62). Previous work has shown that an increase of CD3^neg^CD8α^pos^ NK cells in the ME of PRRSV-infected pregnant gilts can be observed 10 dpi (33). In the current study, however, we did not observe any increase of CD3^neg^CD8α^pos^CD16^pos^CD172a^neg^ NK cells in the ME 21 dpi **(** **Figure 3A** **)**. A plausible explanation for that might be that between 10 and 21 dpi a shift from innate to adaptive responses may have occurred. Furthermore, we also considered the expression of the activating receptor NKp46 (63) and found an increase of NKp46^pos^ NK cells in the FP from No.Vac_Chall_HV fetuses **(** **Figure 4B** **)**. This increase coincided with a drop in NKp46^neg^ NK cells, which could either be explained by the reacquisition of NKp46 on these cells or the influx of more NKp46^pos^ cells. *In vitro* experiments have demonstrated that NKp46 expression can be induced on sorted NKp46^neg^ NK cells following cytokine stimulation (63). For NKp46^pos^ NK cells in blood and spleen, it has been shown that their capacity to produce cytokines and cytolytic activity is higher compared to NKp46^neg^ NK cells (64). NKp46^high^ expressing NK cells are considered to be superior in context of cytokine production and cytolytic activity (64), but recent data suggests that NKp46 downregulation occurs during porcine NK cell differentiation (Schmuckenschlager et al., manuscript in preparation). In addition, we have also demonstrated that all NK cells at the maternal-fetal interface contain perforin (37). Therefore, it seems likely that the NK cells in the FP are combatting the virus. Further investigations are needed to prove this hypothesis.

The exact role of γδ T cells in context of PRRSV infection is not fully understood. In this study, total γδ T cells were significantly lower at the maternal-fetal interface of No.Vac_Chall_HV fetuses as compared to the Vac_Chall_HV and No.Vac_No.Chall fetuses **(** **Figure 3** **)**. Moreover, in the FP of No.Vac_Chall_HV fetuses, there was a significant change towards a dominance of CD8α^high^ γδ T cells at the expense of the CD8α^neg/dim^ γδ T cells **(** **Figure 5B** **)**. Based on our previous data, where CD8α expression was mainly associated with a CD2^pos^ phenotype (37), we presume that the CD8α^high^ and CD8α^neg/dim^ γδ T closely correspond to a CD2^pos^ and CD2^neg^ phenotype, respectively. Distinct cytokine production profiles have been associated with the two γδ T cell subsets (65). A CD2^pos^ phenotype is associated with a higher capacity to produce IFN-γ (65), and exclusively expresses perforin (66). The latter was also demonstrated for CD2^pos^ γδ T cells at the maternal-fetal interface (37). This suggests that the identified increase in CD8α^high^ γδ T cells in the FP might have exhibited inflammatory and potentially cytotoxic functions in No.Vac_Chall_HV fetuses.

CD4 T cells can promote the B cell and CD8 T cell function in context of antiviral immunity (67). In the current study, the CD8α/CD27-expression pattern was used to assess CD8α^pos^CD27^pos^ early effector or central memory (Tcm) and CD8α^pos^CD27^neg^ late effector or effector memory phenotype at the maternal-fetal interface. A clear increase in CD4 T cells with an early effector phenotype was observed for No.Vac_Chall_HV fetuses, and coincided with a drop of CD8α^neg^CD27^pos^ naive CD4 T cells **(** **Figure 6** **)**. It seems that this increase in early effector T cells is a response to HV PRRSV infection. However, further functional characteristics and PRRSV-specificity of CD4 T cells need to be characterized. CD8 T cells are important components of the adaptive immune system responsible for the elimination of virus-infected cells. CD8β-expressing T cells with a putative CD8α^pos^CD27^dim^ early effector phenotype were the main responders at the maternal-fetal interface of No.Vac_Chall_HV and No.Vac_Chall_LV fetuses **(** **Figure 7** **)**. Furthermore, our previous work has shown that CD8β T cells with an early effector phenotype readily express perforin (37), which is indicative of a cytotoxic potential. Overall, research addressing local CD8 T cell responses is limited. Previously, it has been shown that peripheral blood CD8 T cells, isolated 21 dpi, readily proliferate upon restimulation *in vitro* (68). However their capacity to kill PRRSV-infected macrophages only occurred 49 dpi (68). Recent work has shown that CD8 T cells might play a pivotal role at the site of infection, particularly in lung and brochoalveolar lavage (20, 69). Future work is needed to address the PRRSV-specific CD8 T cell responses and their functional capacity *in utero*.

Hence, the results of our study indicate that the HV PRRSV-1 field isolate causes an influx of early effector phenotypes at the maternal-fetal interface, including NKp46^pos^ NK cells, CD8α^high^ γδ T cells, as well as CD8α^pos^CD27^pos/dim^ CD4 and CD8 T cells. We postulate that this substantial increase in effector phenotypes is an indicator of local tissue damage potentially resulting in focal detachment of the placenta and consequently fetal demise. Of note, in the ME of vaccinated gilts (*e.g.* Vac_Chall_LV and Vac_Chall_HV), this increase of CD4 and CD8 early effector T cell phenotypes compared to No.Vac_No.Chall and Vac_No.Chall groups was more contained. This may suggest that the challenge infection lead to a re-activation of pre-existing memory T cells, induced by the MLV vaccine, that was “just about right” to control viral replication yet avoided an excessive inflammatory response. However, depending on the PRRSV field strain and response to vaccination, in some gilts/sows the local response might not be sufficient to prevent vertical transmission (as observed in gilt #15, **Supplementary Figure 2**).

In conclusion, using flow cytometry, we have shown that PRRSV induces changes in immune cell phenotypes that reside at the maternal-fetal interface. Our study suggests that the local activation of effector phenotypes in response to high-virulent PRRSV strains might cause immune-pathogenesis, as the result of local inflammation, apoptosis and bystander apoptosis, causing focal detachment of the maternal-fetal interface, contributing to reproductive failure. In addition, our data indicates that vaccination by MLVs may limit such local immune activation with potentially beneficial or detrimental consequences. However, functional aspects of the addressed immune cell phenotypes need further investigation, as it is assumed that PRRSV utilizes various immune modulatory mechanisms (5, 70–72).

## Data availability statement

The original contributions presented in the study are included in the article/**Supplementary Material**. Further inquiries can be directed to the corresponding author.

## Ethics statement

The animal study was reviewed and approved by institutional ethics and animal welfare committee (Vetmeduni Vienna) and the national authority according to §§26ff. of Animal Experiments Act, Tierversuchsgesetz 2012 – TVG 2012 (GZ 68.205/0142-WF/V/3b/2016).

## Author contributions

MRS, KM, JS, AS, TR, WG, and AL were in charge of the study design. HK, CK, JS, GB, and AL organised the animal experiment and were responsible for the sample collection. ES, KM, MK, MS, MZ, MM, and TR performed the laboratory work. MRS, WG, and AL analysed the data and MD performed the statistical analysis. MRS, WG, and AL discussed and interpreted the data and prepared the manuscript. All authors read and approved the final manuscript.

## Funding

The authors declare that this research was funded by Boehringer Ingelheim Vetmedica GmbH. However, the funder was not involved in the study design, data analysis and interpretation, the writing process, or the decision to submit the manuscript for publication. GB was supported by the János Bolyai Research Scholarship of the Hungarian Academy of Sciences.

## Acknowledgments

We would like to express our gratitude to all people involved in the animal experiment, particularly during sample collection at necropsy and sample processing in the lab. In addition, we would also like to thank Simona Winkler for providing us with the immunofluorescence microscopy picture.

## Conflict of interest

The authors declare that the research was conducted in the absence of any commercial or financial relationships that could be construed as a potential conflict of interest.

## Publisher’s note

All claims expressed in this article are solely those of the authors and do not necessarily represent those of their affiliated organizations, or those of the publisher, the editors and the reviewers. Any product that may be evaluated in this article, or claim that may be made by its manufacturer, is not guaranteed or endorsed by the publisher.
