# Supplementary material for: Influence of PRRSV-1 vaccination and infection on mononuclear immune cells at the maternal-fetal interface
Source: Front Immunol. 2022 Nov 8;13:1055048. doi: 10.3389/fimmu.2022.1055048 (PMC9679432; doi:10.3389/fimmu.2022.1055048)

Supplementary Material

Influence of PRRSV-1 vaccination and infection on mononuclear immune cells at the maternal-fetal interface

**Melissa R Stas^1^, Heinrich Kreutzmann^1^, Julia Stadler^2^, Elena L Sassu^1,¥^, Kerstin H Mair^3,4^, Michaela Koch^1^, Christian Knecht^1^, Maria Stadler^3^, Marlies Dolezal^5^, Gyula Balka^6^, Marianne Zaruba^7^, Marlene Mötz^7^,Armin Saalmüller^3^, Till Rümenapf^7^, Wilhelm Gerner^3,§,Ŧ^, and Andrea Ladinig^1,Ŧ^**

Correspondence: Andrea Ladinig ([Andrea.ladinig@vetmeduni.ac.at](mailto:Andrea.ladinig@vetmeduni.ac.at))
Ŧ These authors have contributed equally to this work

# Supplementary Figures

**Supplementary Figure 1 | Consecutive gating strategy applied for the identification of lymphocytes at the maternal-fetal interface.** For the characterization of the major lymphocyte subsets a similar gating hierarchy was applied. Each individual sample was inspected using the time parameter in order to ensure a stable flow of cells was established. A time gate was applied in order to exclude areas with a poor flow (e.g. due to a clog or an air bubble). Hereafter, lymphocytes and larger leukocytes were identified according to their light scatter properties (forward scatter area (FSC-A) vs. side scatter area (SSC-A) and a double doublet discrimination (forward scatter area (FSC-A) vs. forward scatter height (FSC-H) and side scatter (SSC-A) vs. side scatter height (SSC-H)) was performed. Cells with high autofluorescence were excluded using the 510/50 bandpass filter in the excitation line of the violet laser and dead cells were excluded based on the staining with the fixable viability dye eFluor780^®^. Firstly, total CD45^pos^ cells within viable lymphocytes were identified (black box) using the same lymphocyte identification gate for each individual sample. The frequency obtained for total CD45^pos^ cells was used to calculate the CD45 correction factor to correct the obtained cell lineage frequencies. Major lymphocyte subsets were identified within viable lymphocytes. B cells were gated based on their expression of CD79α. The T cell subsets were gated based on their expression of TCR-γδ, CD4, and CD8β for the identification of total γδ T cells, CD4 T cells, and CD8β T cells, respectively. For the characterization of NK cells, CD16^pos^CD172a^neg^ cells were selected and sub-gated for a CD3^neg^CD8α^pos^ phenotype. Myeloid cells were identified based upon their CD172a expression. Representative pseudocolor plots for their hierarchical structure is shown for the maternal endometrium (ME) in **(A)** and fetal placenta (FP) in **(B)** from a No.Vac_No.Chall fetus.


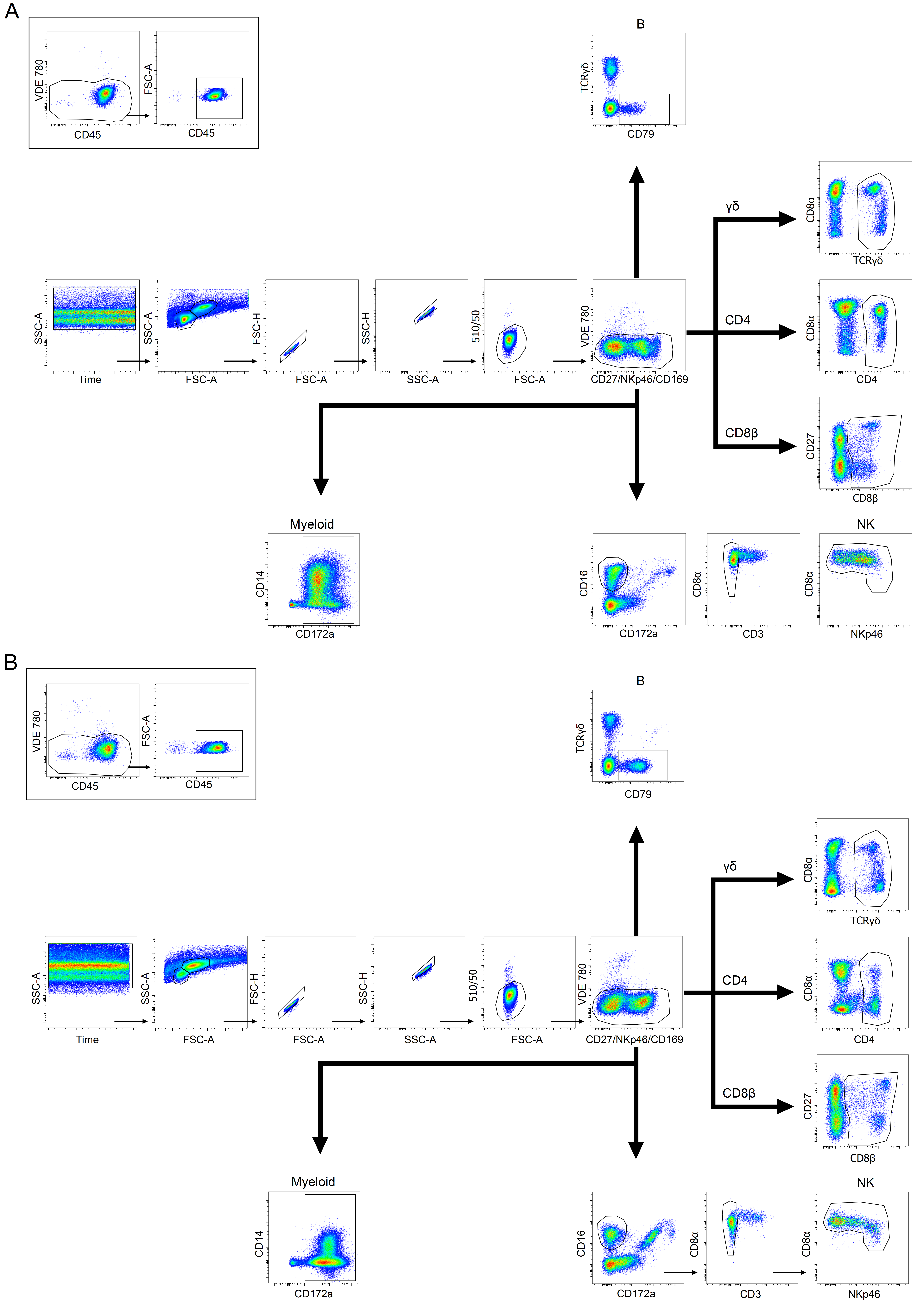


**Supplementary Figure 2 | Fetal preservation status and viral load at the maternal-fetal interface of individual fetuses.** Following the opening of the uteri, the position of each fetus, within the left and right uterine horn, was recorded and the fetal preservation status was evaluated. The categories for the fetal preservation status included VIA, MEC, DEC, and AUT in order to identify the viable, meconium stained, decomposed, and autolyzed fetuses, respectively. The viral load for the maternal endometrium (ME) and fetal placenta (FP) for each individual fetus was determined using an ORF7 PRRSV-1 isolate specific RT-qPCR. The results for the viral load are given as genome equivalents per gram tissue (log_10_ transformed). The color codes indicate the magnitude of the viral load. (*) means below the detection limit and (-) sample was not taken. Within one color coded frame (e.g. No.Vac_Chall_LV), each line depicts the results for the fetal preservation status, viral load in the ME, and viral load in the FP for one litter. The first column always indicates the gilt ID. Fetuses used for *ex vivo* phenotyping are indicated in bold and blue (section of fetal preservation). The depicted treatment groups are: No.Vac_Chall_LV (dark purple, non-vaccinated and infected low virulent strain), Vac_Chall_LV (light purple, vaccinated and infected low virulent strain), No.Vac_Chall_HV (dark red, non-vaccinated and infected high virulent strain), and Vac_Chall_HV (light red, vaccinated and infected high virulent strain).


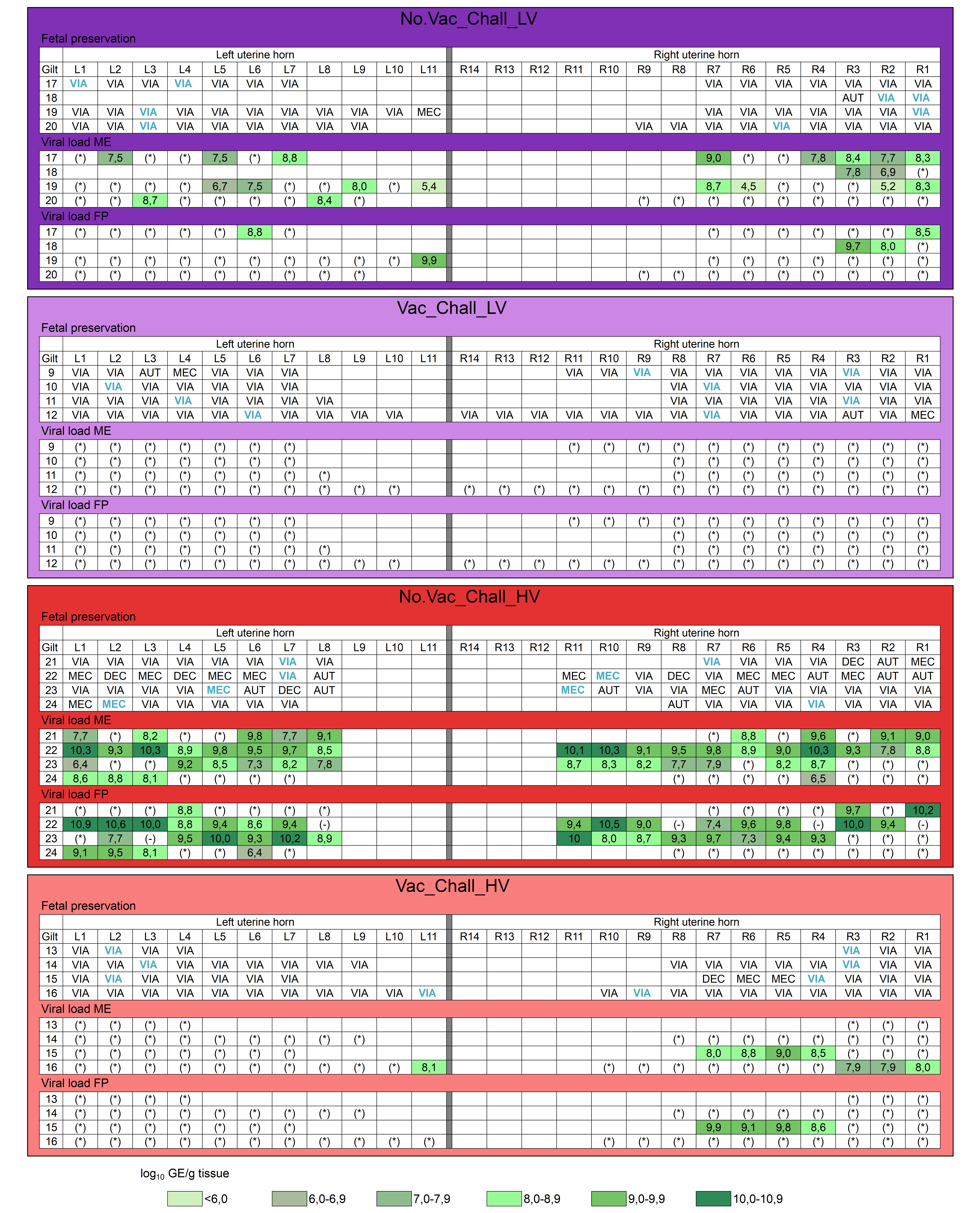


**Supplementary Figure 3 | Detection of porcine reproductive and respiratory syndrome virus at the maternal-fetal interface using immunofluorescence.** Cryopreserved tissue from a No.Vac_Chall_HV fetus was sectioned (6 µm thick) and immunofluorescence was performed. A staining for nuclei (DAPI, blue), PRRSV-NP (IgG2a, clone P11/d72-c1, green), and cytokeratin-8 ((CK8), TROMA-1, grey) was applied. Stained sections were scanned using an Axioimager Z.1 wide field microscope (Zeiss, Germany) equipped with a mercury lamp, using a LD Plan-Neofluar 20x/0.40 air NA lens coupled to an ultra-compact 14 bit CCD camera (PCO PixelFlyUSB, PCO AG, Kelheim, Germany) to obtain images. For the detection of DAPI, PRRSV-NP, and CK8 the exposure time was set to 80 ms for all three channels and signal was detected using the DAPI, GFP and Cy5 filters, respectively. CK8, strongly stains the endometrial epithelium and was used to identify the maternal and fetal boundaries of the fetal-maternal junction (FMJ). The maternal endometrium (ME) and fetal placenta (FP) were identified. Yellow arrowheads indicate viral antigen, which is predominantly located in the FP, in close proximity to the FMJ. MSF = maternal secondary fold, UG = uterine glands. Scale bar = 200 µm.


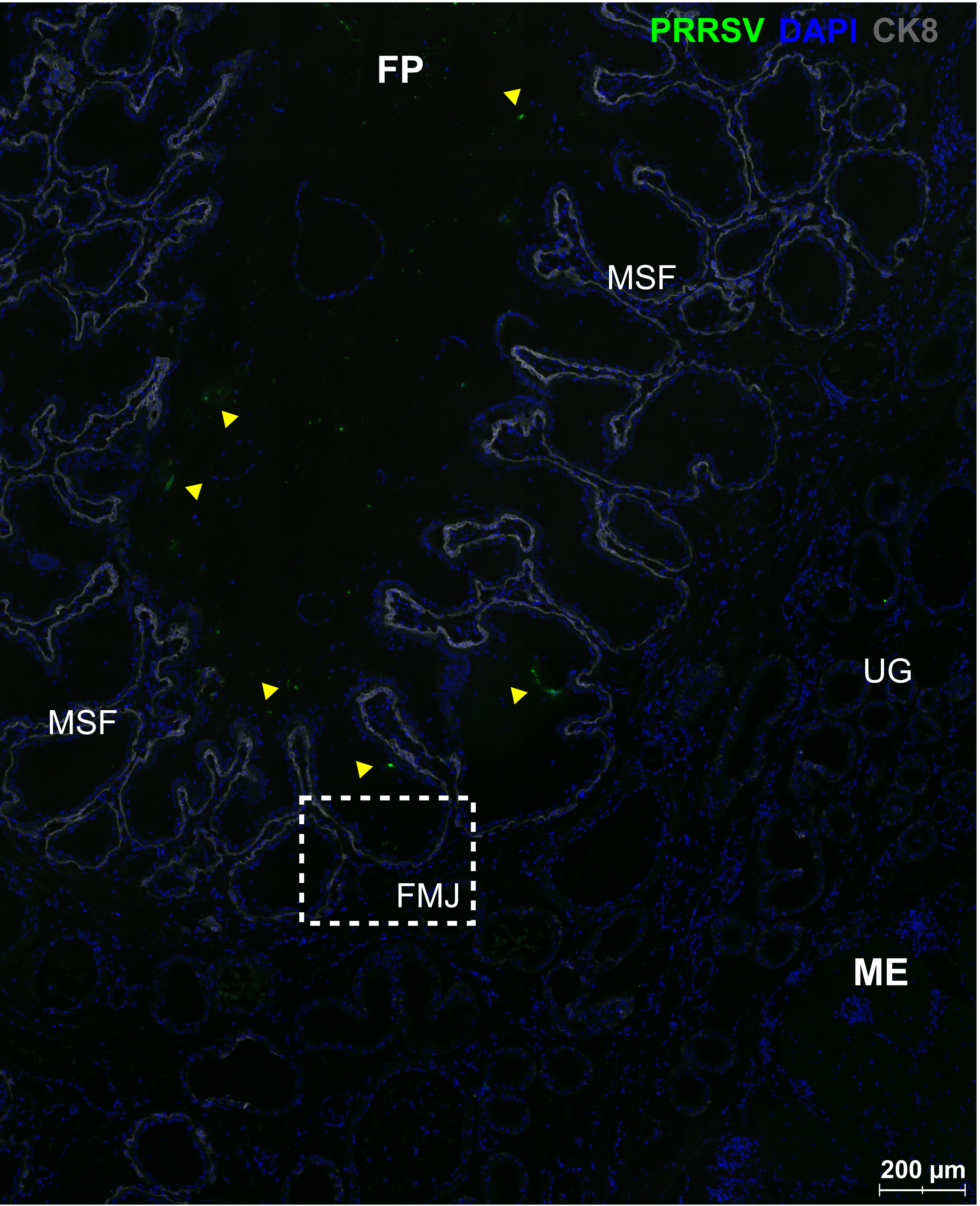

Supplement: Supplementary file 1 [file DataSheet_1.docx]
